# A Case of Acute Myocardial Infarction in a Patient Whose Initial Complaints Were Hematemesis and Epigastric Discomfort

**DOI:** 10.1155/2019/5984251

**Published:** 2019-05-23

**Authors:** Kazuhiko Omori, Youichi Yanagawa

**Affiliations:** Department of Acute Critical Care Medicine, Shizuoka Hospital, Juntendo University, Japan

## Abstract

The patient was a 64-year-old woman with systemic lupus erythematosus, thrombophlebitis of the lower legs, cerebral infarction with left hemiparesis, and colostomy after perforation of the sigmoid colon. On the morning of her presentation, the patient felt epigastric abnormality. Thereafter, hematemesis occurred twice, leading her to call an ambulance in the afternoon. Upon arrival, electrocardiography before securing a venous route and obtaining blood samples revealed ST segment elevation in leads II, III, and aVF. As her vital signs were stable and her hemoglobin level had decreased by just 1.1 g/dl in comparison to the previous day, emergency coronary angiography (CAG) was performed. CAG revealed complete occlusion at section #4. She underwent right coronary angioplasty with stent placement. The patient's course after angioplasty was uneventful. On the 15^th^ hospital day, esophagogastroduodenoscopy revealed esophageal erosion and superficial gastritis. She was discharged on foot the following day. When physicians treat patients with hematemesis, electrocardiography and the measurement of troponin are essential before esophagogastroduodenoscopy.

## 1. Introduction

Complication between gastroduodenal bleeding and acute coronary syndrome is not uncommon [1–4]. We report the case of a patient with acute myocardial infarction whose initial complaints were hematemesis and epigastric discomfort.

## 2. Case Report

**Figure 1:**
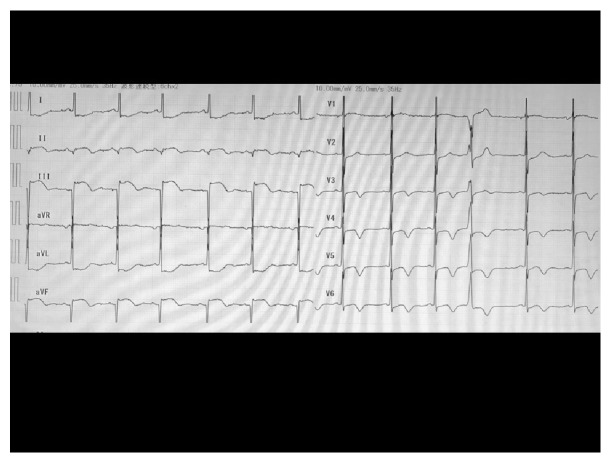
Electrocardiography on arrival. The electrocardiogram showed ST segment elevation in leads II, III and aVF.

**Figure 2:**
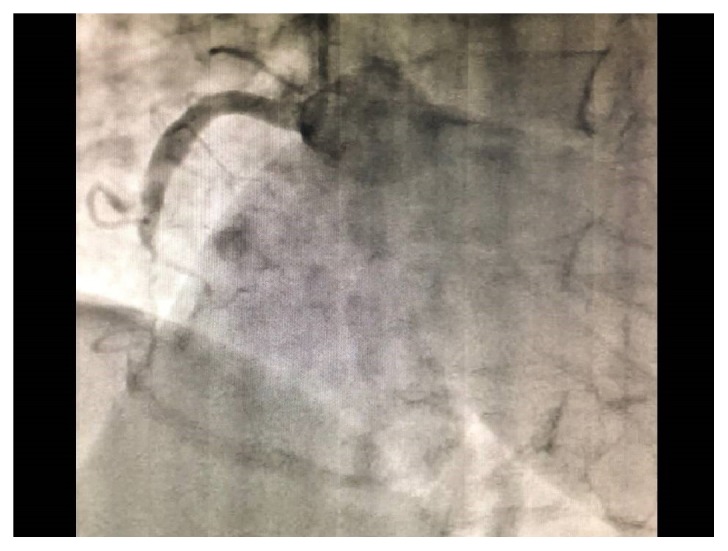
Emergency coronary angiography (CAG). CAG demonstrated 99% stenosis at section #2, and complete occlusion at section #4.

**Figure 3:**
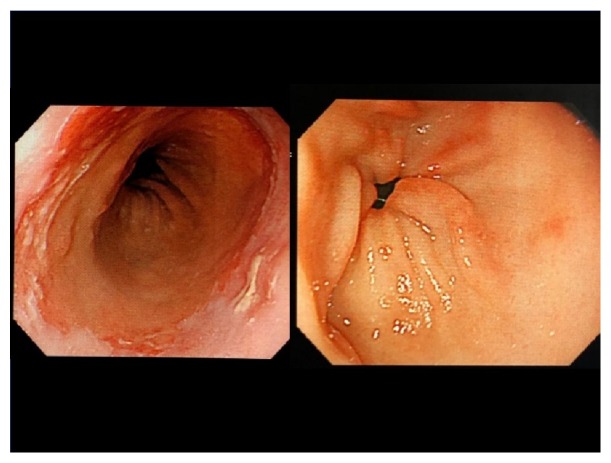
Esophagogastroduodenoscopy on the 15^th^ hospital day. Esophagogastroduodenoscopy revealed an esophageal erosion (left) and superficial gastritis (right).

The patient was a 64-year-old woman with systemic lupus erythematosus, thrombophlebitis of the lower legs, cerebral infarction with left hemiparesis, and colostomy after perforation of the sigmoid colon. She was treated with prednisolone, tacrolimus, mizoribine, edoxaban, limaprost, famotidine, sulfamethoxazole-trimethoprim, sertraline, eszopiclone, and minodronic. On the morning of her presentation, the patient felt epigastric abnormality. Thereafter, hematemesis occurred twice, leading her to call an ambulance in the afternoon. Upon arrival, her vital signs were as follows: Glasgow Coma Scale, E4V5M6; blood pressure, 110/76 mmHg; pulse rate, 78 beats per minute; and her peripheral oxygen saturation on 6 liters of oxygen per minute, 98%. A physiological examination revealed preexisting bilateral leg edema with pigmentation and left hemiparesis. Electrocardiography before securing venous route and blood examination revealed ST segment elevation in leads II, III, and aVF (Figure 1). Chest roentgenography showed cardiomegaly and cardiac ultrasound showed hypokinesis at the inferior wall. The results of a biochemical blood analysis on arrival were as follows: white blood cell count, 11,500/*μ*L; hemoglobin, 9.6 g/dL; platelet count, 16.8 ×10^4^/*μ*L; total protein, 6.1 g/dL; total bilirubin, 0.5 mg/dL; aspartate aminotransferase, 86 IU/L; alanine aminotransferase, 8 IU/L; blood urea nitrogen, 13.7 mg/dL; creatinine, 0.49 mg/dL; sodium, 143mEq/L; potassium, 3.6mEq/L; chloride, 106mEq/L; creatine phosphokinase, 1000 IU/L; troponin T, 13250 (26.2 >) pg/mL; prothrombin time, 12.7 (11.7) s; activated partial thromboplastin time, 30.1 (30.2) s; fibrinogen, 326 mg/dL; and D-dimmer, 0.79*μ*g/mL. She was diagnosed with acute myocardial infarction with upper esophagogastroduodenal bleeding. As her vital signs were stable and her level of hemoglobin decreased by just 1.1 g/dl in comparison to the previous day when she had visited the dermatology department of Numazu City Hospital, she underwent emergency coronary angiography (CAG). CAG demonstrated 99% stenosis at section #2, complete occlusion at section #4 (Figure 2), and 75% stenosis at section #6. She underwent right coronary angioplasty with stent placement. The prescriptions of edoxaban and sertraline were stopped, famotidine was switched to lansoprazole, and treatment with aspirin, clopidogrel, rosuvastatin, and carvedilol was initiated. After angioplasty, her course was uneventful. Her creatinine kinase level peaked at 5655 IU/L on the 2^nd^ hospital day, and her minimum level of hemoglobin was 8.3 g/dl on the 7^th^ hospital day without transfusion. On the 15^th^ hospital day, esophagogastroduodenoscopy revealed esophageal erosion and superficial gastritis (Figure 3). She was discharged on foot the following day.

## 3. Discussion

Esophagogastroduodenoscopy revealed esophageal erosion and gastritis; thus, we hypothesized that the patient's initial epigastric discomfort was induced by acute myocardial infarction, following stress-induced esophageal erosion and gastritis. However, it is possible that preexisting bleeding from esophagogastric lesions induced acute myocardial infraction due to a mismatch from decreasing oxygen transport ability and increased cardiac oxygen consumption capacity due to anemia.

The typical complaint of patients with acute myocardial infarction is chest pain; however, acute myocardial infarction is also associated with atypical complaints or signs such as left shoulder pain, pharyngeal pain, earache, headache, back pain, syncope, and dyspnea. In cases involving atypical complaints, signs, or symptoms, the diagnosis and treatment tend to be delayed, resulting in an unfavorable outcome [5, 6]. It is not uncommon for patients with acute myocardial infarction to be complicated by esophagogastroduodenal lesions or patients with esophagogastroduodenal lesions to be complicated by acute myocardial infarction. Accordingly, when physicians treat such patients, both possibilities should be excluded as soon as possible. For example, in case of patients with hematemesis, it is essential to perform electrocardiography and measure the troponin level before esophagogastroduodenoscopy, similarly to our case.

The optimal treatment strategy for the combined acute myocardial infarction and hematemesis is controversial because the standard treatments for these diseases are totally different [7]. Acute myocardial infarction requires antiplatelet therapy, which may lead to the deterioration of hematemesis. In contrast, hematemesis requires treatment to achieve hemostasis, which may lead to the deterioration of acute myocardial infarction. Accordingly, these complications can lead to unfavorable outcomes. Our treatment strategy fortunately resulted in a favorable outcome; however, further examinations are needed to select appropriate treatments for patients with the combination of acute myocardial infarction and hematemesis.

## References

[1] Mumtaz K., Wasim F., Jafri W. (2008). Safety and utility of oesophago-gastro-duodenoscopy in acute myocardial infarction. *European Journal of Gastroenterology & Hepatology*.

[2] Lin S., Konstance R., Jollis J., Fisher D. A. (2006). The utility of upper endoscopy in patients with concomitant upper gastrointestinal bleeding and acute myocardial infarction. *Digestive Diseases and Sciences*.

[3] Bhatti N., Amoateng-Adjepong Y., Qamar A., Manthous C. A. (1998). Myocardial infarction in critically ill patients presenting with gastrointestinal hemorrhage: Retrospective analysis of risks and outcome. *Chest*.

[4] Prendergast H. M., Sloan E. P., Cumpston K., Schlichting A. B. (2005). Myocardial infarction and cardiac complications in emergency department patients admitted to the intensive care unit with gastrointestinal hemorrhage. *The Journal of Emergency Medicine*.

[5] Roach L. (2010). Treating patients with atypical cardiac presentations recognize & treat everyday cardiac complaints. *Journal of Emergency Medical Services*.

[6] Hwang S. Y., Ahn Y. G., Jeong M. H. (2012). Atypical symptom cluster predicts a higher mortality in patients with first-time acute myocardial infarction. *Korean Circulation Journal*.

[7] Ergelen M., Uyarel H., Soylu Ö. (2010). Gastrointestinal bleeding in patients undergoing primary angioplasty for acute myocardial infarction: Incidence, risk factors and prognosis. *Türk Kardiyoloji Derneği Arşivi*.

